# Breastfeeding and allergic disease at age nine in Ireland: An Irish prospective cohort study

**DOI:** 10.1093/eurpub/ckac130.246

**Published:** 2022-10-25

**Authors:** E Cosgrave, P Fitzpatrick

**Affiliations:** 1 Department of Public Health HSE South East, Health Service Executive, Kilkenny, Ireland; 2 School of Public Health, University College Dublin, Dublin, Ireland

## Abstract

**Background:**

Despite well-recognised benefits, Irish breastfeeding rates remain suboptimal. Although associations between breastfeeding and allergic disease are well-researched in younger children, evidence for continued effect in older children is sparse. This Irish prospective cohort study investigated associations between breastfeeding and allergic disease at age nine.

**Methods:**

The study sample included all nine-year-old children enrolled in the Growing Up in Ireland Infant Cohort Study whose mothers had participated in both Wave 1 and Wave 5. Mothers self-reported infant feeding practices at nine months and allergic diseases at nine years. Multiple logistic regression was used to generate adjusted odds ratios (aOR) for associations between breastfeeding and allergic diseases; re-weighting was applied to enhance generalisability.

**Results:**

Response rate was 72% (N = 8,006). Most mothers (53%) had ever-breastfed their child; younger mothers, smokers and those of lower socioeconomic status were significantly less likely to have ever-breastfed. Compared to never-breastfeeding, ever-breastfeeding was protective against asthma (aOR 0.74, 95%CI 0.62-0.87) and eczema (aOR 0.72, 95%CI 0.55-0.93) at age nine. Ever-breastfeeding increased the risk of atopic rhinitis (aOR 1.44, 95%CI 1.07-1.94); the association with food allergy was inconclusive (aOR 1.17, 95%CI 0.83-1.64). Breastfeeding ≥6 months was protective against asthma (aOR 0.57, 95%CI 0.39-0.82) and any allergic disease (aOR 0.72, 95%CI 0.55-0.96). Exclusive breastfeeding (3-5 months) was protective against asthma (aOR 0.67, 95%CI 0.50-0.89) and eczema (aOR 0.54, 95%CI 0.34-0.86).

**Conclusions:**

This study provides new evidence suggesting breastfeeding may be protective against asthma and eczema but may increase the risk of atopic rhinitis in older Irish children. Results must be considered in light of high Irish allergic disease prevalence and action in support of breastfeeding prior to and following birth prioritised accordingly.

**Key messages:**

- Findings lend support to a protective association between breastfeeding and asthma and eczema in later childhood and indicate breastfeeding may play a role in allergic disease prevention.
- Breastfeeding may reduce the risk of asthma and eczema in Irish children: findings should be used to drive impactful breastfeeding promotion and reorientate cultural norms in favour of breastfeeding.

